# S100A9 Induced Inflammatory Responses Are Mediated by Distinct Damage Associated Molecular Patterns (DAMP) Receptors *In Vitro* and *In Vivo*

**DOI:** 10.1371/journal.pone.0115828

**Published:** 2015-02-23

**Authors:** Bo Chen, Allison L. Miller, Marlon Rebelatto, Yambasu Brewah, Daniel C. Rowe, Lori Clarke, Meggan Czapiga, Kim Rosenthal, Tomozumi Imamichi, Yan Chen, Chew-Shun Chang, Partha S. Chowdhury, Brian Naiman, Yue Wang, De Yang, Alison A. Humbles, Ronald Herbst, Gary P. Sims

**Affiliations:** 1 MedImmune LLC, One MedImmune Way, Gaithersburg, Maryland 20878, United States of America; 2 Laboratory of Human Retrovirology, Applied and Developmental Directorate, Building 550 Room 126, Leidos Biomedical Research Inc, Frederick National Laboratory for Cancer Research, Frederick, Maryland 21702, United States of America; 3 Laboratory of Molecular Immunoregulation, National Cancer Institute, Frederick National Laboratory for Cancer Research, Frederick, Maryland 21702, United States of America; University of Pittsburgh, UNITED STATES

## Abstract

Release of endogenous damage associated molecular patterns (DAMPs), including members of the S100 family, are associated with infection, cellular stress, tissue damage and cancer. The extracellular functions of this family of calcium binding proteins, particularly S100A8, S100A9 and S100A12, are being delineated. They appear to mediate their functions via receptor for advanced glycation endproducts (RAGE) or TLR4, but there remains considerable uncertainty over the relative physiological roles of these DAMPs and their pattern recognition receptors. In this study, we surveyed the capacity of S100 proteins to induce proinflammatory cytokines and cell migration, and the contribution RAGE and TLR4 to mediate these responses *in vitro*. Using adenoviral delivery of murine S100A9, we also examined the potential for S100A9 homodimers to trigger lung inflammation *in vivo*. S100A8, S100A9 and S100A12, but not the S100A8/A9 heterodimer, induced modest levels of TLR4-mediated cytokine production from human PBMC. In contrast, for most S100s including S100A9, RAGE blockade inhibited S100-mediated cell migration of THP1 cells and major leukocyte populations, whereas TLR4-blockade had no effect. Intranasal administration of murine S100A9 adenovirus induced a specific, time-dependent predominately macrophage infiltration that coincided with elevated S100A9 levels and proinflammatory cytokines in the BAL fluid. Inflammatory cytokines were markedly ablated in the TLR4-defective mice, but unexpectedly the loss of TLR4 signaling or RAGE-deficiency did not appreciably impact the S100A9-mediated lung pathology or the inflammatory cell infiltrate in the alveolar space. These data demonstrate that physiological levels of S100A9 homodimers can trigger an inflammatory response *in vivo*, and despite the capacity of RAGE and TLR4 blockade to inhibit responses *in vitro*, the response is predominately independent of both these receptors.

## Introduction

Sensing pathogens through the recognition of pathogen-associated molecular patterns (PAMPs) by cell surface and intracellular pattern recognition receptors is important in mounting an effective immune response. The innate immune system has also evolved to recognize and respond to endogenous danger signals or danger associated molecular patterns (DAMPs), which may be released by stressed, dying or necrotic cells [1]. Liberation of the archetypal endogenous DAMP, the non-histone DNA binding molecule High Mobility Group Box 1 (HMGB1), has been shown to play a critical role in a variety of inflammatory disorders including sepsis, trauma, cancer and autoimmunity [2,3]. These functions are predominately mediated through the interactions with TLR4 and Receptor for Advanced Glycation Endproducts (RAGE), although direct and indirect interactions with other receptors have also been described. There is mounting evidence that S100 proteins also act as DAMPs and play a similar role in regulating tissue inflammation and injury through the same receptors.

Members of the S100 family are small, acidic calcium binding proteins that are characterized by a C-terminal ‘canonical’ EF-hand calcium binding motif and an N-terminal ‘pseudo’ EF hand motif that are connected by a central hinge region [4]. In humans, over twenty S100 proteins have been identified that exhibit differential tissue and cell-type expression profiles [5]. The structural homology of the S100s permits the formation of active homodimers and heterodimers, as well as higher-order multimeric structures that can alter binding properties of and physiological responses to these proteins [5]. Besides regulating a diverse set of intracellular processes in response to increases in intracellular calcium, there is now increasing interest in the extracellular functions of S100 family members. Initially, S100s were shown to have anti-microbial activities, and were capable of blocking bacterial adherence to mucosal epithelium [6,7]. Subsequently, it was shown that S100 proteins released from different cell types during inflammation serve as useful markers of disease activity for a variety of indications including chronic obstructive pulmonary disease, asthma, rheumatoid arthritis, colitis, Alzheimer’s disease and cancer among others [8–10].

Studies have begun to unravel how individual extracellular S100 proteins may regulate aspects of inflammatory responses. A subset of S100s known as the calgranulins, specifically S100A8 (Calgranulin A, MRP8), S100A9 (Calgranulin B, MRP14) and S100A12 (Calgranulin C, EN-RAGE), are constitutively expressed at high levels in neutrophils and monocytes and can be induced from endothelial and epithelial cells. The earliest studies indicated that S100A12 could trigger cytokines, cellular activation responses *in vitro* and *in vivo* and these effects were mediated by RAGE [11]. S100A12 promotes the adhesion and transendothelial migration by inducing the upregulation of ICAM and VCAM on endothelial cells in a RAGE-dependent manner [11,12]. RAGE is expressed at high levels on lung epithelial cells and at low levels on most leukocytes and endothelial cells. It is upregulated by its ligands and other inflammatory mediators, and binds different classes of endogenous molecules released during cellular or physiological stresses [3]. RAGE also directly interacts with several S100 proteins, including S100A8 and S100A9, as well as the β2 integrin Mac-1 [13,14], and can play an important role in mediating the functions of these proteins [9].

S100A8 and S100A9 are less stable than S100A8/A9 heterodimers and consequently the proinflammatory activities of S100A8 and S100A9 are usually attributed to the heterodimer. However, the formation of a heterodimer is not necessary to induce inflammatory responses, and it was recently shown that the S100A9 homodimers generated under inflammatory conditions are resistant to proteolytic cleavage [15]. It is also noteworthy that assays to measure the levels of S100A8, S100A9 and the heterodimer are not standardized or reported consistently, so although it is clear the overall levels of S100A8 and S100A9 are increased in fluids obtained from patient samples, the relative levels and roles of the homodimers and heterodimers during inflammatory responses remain uncertain.

*In vivo*, our understanding of the extracellular functions has also been limited in part because S100A8 knock-out mice are embryonically lethal, S100A9 knock-out mice, although viable, fail to express S100A8 protein, and the relative importance of the extracellular and intracellular functions of S100A9 cannot be delineated [16,17]. Nevertheless, it is clear that S100A8 and S100A9 are important regulators of immune responses. S100A8, S100A9 or monosodium urate crystals loaded air-pouches induced the migration of neutrophils which could be inhibited with polyclonal anti-S100A8 and anti-S100A9 antibodies [18,19]. Neutralizing S100A8 and S100A9 antibodies also blocked phagocyte migration to the alveoli following Streptococcal pneumonia infection [20]. S100A9 also regulates the accumulation of myeloid-derived suppressor cells in cancer [21]. Subsequently, S100A8 was shown to induce proinflammatory cytokines via TLR4/MD2 *in vitro* [22]. More recent studies indicate that S100A9 and S100A12 may also induce cytokines via TLR4 [23–25]. *In vivo* studies using S100A9-deficient mice, which fail to induce S100A8 or S100A9, were partially protected from endotoxemia and this appeared to be mediated by TLR4 [22], although, an alternate study showed S100A8 administration attenuated endotoxemia mediated inflammation and tissue injury suggesting a protective role for S100A8 [26]. S100A8- and S100A9-driven TLR4-signaling has also been implicated in the induction of TH17-dependent development of autoreactive CD8+ cells in a model of systemic autoimmunity [27], and S100A8 activation of TLR4 in the joint promoted the upregulation of activating FcγRs on macrophages and joint inflammation [28]. Quinoline-3-carboxyamides (Q compounds) which modify disease in both animal models and in clinical trials were shown to bind to S100A9, but not S100A8 or the S100A8/A9 heterodimer, and block its interaction with both RAGE and TLR4 [29], and anti-murine S100A9 antibodies also inhibit collagen-induced arthritis, although the receptors responsible for mediating these effects were not delineated [30].

Together these data indicate that endogenous S100 proteins can promote inflammatory responses which appear to be mediated through the pattern recognition receptors RAGE and TLR4. However, most published reports investigated single S100 family members in different assay systems, making broader conclusions and comparisons difficult, and the relative roles of RAGE and TLR4 *in vivo* remain unclear. Herein, we evaluated the *in vitro* cytokine and chemotactic responses of multiple S100s and the roles of their putative receptors RAGE and TLR4. Since S100A9 is associated with acute and chronic inflammation in the airways [31–33], we chose to validate our *in vitro* findings and assess the physiological role of S100A9 homodimers using an adenoviral-murine S100A9-induced lung inflammation model. Our *in vitro* data indicate that most but not all S100s induce migration in a RAGE-dependent manner, whereas the proinflammatory cytokines induced were TLR4-dependent with the notable exception of S100A16. Unexpectedly, our *in vivo* experiments indicate that murine S100A9 is sufficient to induce airway inflammation independent of RAGE and the TLR4-dependent cytokine induction, posing the interesting possibility that unidentified receptor(s) may be responsible for driving S100-mediated inflammation in the lung, and potentially elsewhere.

## Materials and Methods

### Mice, Primary Human Cells and Cell lines

RAGE deficient (*ager-/-)* mice on a C57/B6 background were generated by Taconic Artemis Pharmaceuticals (Cologne, Germany) for MedImmune and have been described elsewhere [34]. C3H/HeOuJ (TLR4-sufficient), and C3H/HeJ mice which have defective TLR4 signaling [35], were purchased from Jackson Laboratories (Bar Harbor, ME). Mice were housed under pathogen-free conditions and were used in experiments at 8–12 weeks of age. All animal experiments were approved by the MedImmune Institutional Animal Use and Care Committee. Human blood from healthy volunteers was obtained with informed consent by venous puncture under MedImmune’s blood donation program. Human monocytic THP-1 and the murine macrophage RAW cell lines were obtained from the American Type Culture Collection (Manassas, VA).

### S100 Reagents

Full length murine S100A9 (Genbank accession NM_009114.2) was synthesized by Invitrogen (San Diego, CA) and cloned into the adenoviral shuttle plasmid pShuttleCMV (AdEasy system, Agilent). The plasmid containing murine S100A9 and the adenoviral genome, pAd S100A9, was generated by recombination in BJ5183-Ad cells (Agilent, Santa Clara, CA). pAdmurine S100A9 was linearized with PacI and transfected into Ad293 cells (Agilent, Santa Clara, CA). After 7 days, the crude viral lysate (CVL) was harvested and amplified on Ad293 cells. Several days later, cytopathic effect (CPE) was seen and the CVL harvested. The CVL was used to infect a large scale culture of 293F cells (Invitrogen, San Diego, CA). Forty-eight hours post infection the virus was harvested and purified on two cesium chloride gradients (one step and one continuous). Expression of murine S100A9 protein was confirmed in the supernatants of MLE infected cells by Western Blot. For mammalian expression of murine S100A9, a 6X His tag was added to the C-terminus of the murine S100A9 in the pShuttleCMV vector. The vector was used for transient expression of HEK293F cells using standard lipid transfection methods, and protein was purified from the supernatant using Nickel columns. Endotoxin levels of purified mS100A9 (<0.001 EU/μg) were determined using Limulus Amebocyte Lysate (LAL) assay (Charles River, Willmington, MA).

Bacterially expressed low endotoxin recombinant human S100A8, S100A9 and S100A12 (endotoxin levels <0.01 EU/μg as determined by manufacturer using LAL assay), along with preparations of S100A1, S100A4, S100A6, S100A7, S100A10, S100A14, S100A16, S100B and S100P (endotoxin levels not specified) were purchased from MBL International (Woburn, MA). Rather than denaturing and renaturing recombinant proteins to generate S100A8/A9 heterodimers, S100A8/A9 heterodimers were purified directly from human neutrophils. Purified neutrophils were suspended in a PBS cocktail of protease inhibitors (Sigma Aldrich, St. Louis, MO) and sonicated for three cycles to obtain a cell lysate. Cytoplasmic fractions were isolated by centrifugation and dialyzed against Buffer A (50 mM Tris HCl (pH8.0) containing 1 mM EDTA, 1 mM DTT, 1 mM CaCl_2_, protease inhibitor cocktail), and captured on a the HiTrap Q HP (GE-Healthcare, Pittsburgh, PA). The bound S100A8/A9 protein was eluted with a 0–50% gradient of Buffer B (Buffer A with 500 mM NaCl), and fractions containing S100A8/A9 proteins were determined by Western blot with anti-S100A8 or S100A9 antibodies (Santa Cruz, CA, USA). Pooled S100A8/A9 fractions were diluted 10-fold with Buffer C (50 mM sodium acetate (pH 4.5), 1 mM EDTA, 1 mM DTT, 1 mM CaCl_2_) and then positive fractions were applied to a HiTrap SP-HP column (GE Healthcare, Pittsburgh, PA). The column was washed with Buffer C containing 300 mM NaCl, and the S1008/9 protein was eluted with an increasing concentration of NaCl (300–500 mM) in Buffer C. The eluted S100A8/A9 fraction was dialyzed against PBS 1 mM CaCl_2_. Contaminating endotoxin was removed by Affinity Pak Detoxi-Gel (Thermo Fisher Scientific Inc, Waltham, MA). The purity of the protein > 95% by SDS-PAGE with endotoxin levels < 0.004 EU/μg protein by LAL assay.

### Ligand blocking antibodies

Murine anti-hTLR4 Ab HTA125 and mIgG2a control Ab (Biolegend, San Diego, CA), rat anti-mTLR4/MD2 Ab MTS510 and rIgG2a control Ab (eBioscience, San Diego, CA), anti-RAGE Ab 4F4 in hIgG1 and mIgG1 formats and respective hIgG1 and mIgG1 control Abs (MedImmune LLC, Gaithersburg, MD) and Polymyxin B (InvivoGen, San Diego, CA) were used at 10 μg/ml unless otherwise stated.

### Cytokine Assays

Human PBMC or murine were stimulated with S100 proteins for 16 h with or without ligand blocking antibodies. LPS and Polymyxin B (InvivoGen, San Diego, CA) were used at 10 ng/ml and 10 μg/ml respectively. Supernatants were collected and IFN-γ, IL-6, IL-1β and TNFα levels were measured using a human or murine proinflammatory cytokine kit (Meso Scale Discovery Inc, Gaithersburg, MD).

### MEK/ERK and PI3K activation assay

THP-1 cells were stimulated with S100A9 protein for 30 min and activation of MEK/ERK and PI3K signaling pathways were measured by using Phospho(Thr202/Tyr204; Thr185/Tyr187)/Total ERK1/2 whole cell lysate kit and Phospho(Ser473)/Total AKT whole lysate kit (Meso Scale Discovery Inc, Gaithersburg, MD).

### Chemotaxis Assay

Cell migration was assessed using a 96-well ChemoTX system (Neuro Probe, Gaithersburg, MD). Human S100 proteins or hMCP-1 (R&D Systems, Minneapolis, MN) were diluted in RPMI 1640 containing 1% BSA and placed in the lower 25 μl chamber. Cells were washed and suspended in the same medium. THP-1 cells, human PBMC or human granulocytes were incubated with or without antibodies for 30 mins at 37°C before the cells were added to the upper chamber. Cells were allowed to migrate for 3 h for PBMC (8 μm filter) and RAW cells (5 μm filter), 1.5 h for THP-1 (5 μm filter) or 1 h for granulocytes (5 μm filter). Migration of cells to the human S100 proteins or human MCP-1 in the lower chamber was enumerated by flow cytometry. The migration index is the ratio of the number of cells that migrate in response to the chemotactic agent versus the number of cells that migrate in its absence. Initial dose range finding assays were used to determine the optimal concentration of each S100 protein. All antibodies used for flow cytometry analysis were purchased from BD/PharMingen (San Diego, CA). PBMCs were incubated with mouse anti-human CD3-PeCy7, CD4-Pacific orange, CD8-Alexa Fluor 488, CD14-PE, CD11B- Alexa Fluor 700, CD19-APC-Cy7 and CD56-APC antibodies 4 degree for 30 min, after wash with FACS buffer (PBS, 1% FBS, pH 7.4), cells were analyzed with a BD LSR II Flow Cytometer (Becton Dickinson, San Jose,CA). Granulocytes were gated by forward and side scatter. For some chemotaxis experiments cells were preincubated for 30 mins with small molecule inhibitors PD98059, U0126, Sb203580, wortmannin, ly294002 (Cell signaling Technology, Danvers, MA) and BAY11-7082 (Sigma-Aldrich, St Louis, MO) or vehicle control. THP-1 cells (10^6^/sample) were treated with small molecule inhibitors for 6 h, cell viability was measured by using Vybrant Apoptosis Assay kit #2 (Invitrogen, Carlsbad CA) using the protocols provided by the manufacturer.

### Adeno-S100A9 infection and necropsy of mice

Mice were anesthetized with isofluorane prior to intranasal administration of 50 μl of PBS, or 3x10^8^ pfu of Adenovirus null (Adeno-null) or Adenovirus expressing murine S100A9 (Adeno-mS100A9) in PBS. At the time of necropsy we collected either BAL fluid (obtained by 3 x 0.6 ml washes with PBS/10mM EDTA/20mM Hepes) for cell counts and cytokine analyses, or lung tissue for histopathology assessment.

### BAL fluid analyses

Cells recovered from the BAL fluids were cytospun onto glass slides and were stained with diff-quik to allow for identification of leukocyte populations. A total of 500 cells were counted for each sample and percentages of eosinophils, macrophages, lymphocytes and neutrophils were calculated. Percentages were used to back-calculate to total numbers for each cell type. Cytokines were measured using the mouse proinflammatory cytokines kit (Meso Scale Discovery Inc, Gaithersburg, MD). Murine S100A9 was detected by western blot using anti-S100A9 antibody (R&D systems, Minneapolis, MN).

### Histopathology

Lung samples were fixed in 10% buffered formalin for at least 24 h. For histopathology 5 μm thick sections were cut from paraffin blocks and mounted on SuperFrost Plus slides. Slides were stained with hematoxylin and eosin (H&E). Assessment of tissue pathology was performed by a trained pathologist, blinded to the individual samples, using a 0–5 scoring system: 0—no to rare inflammatory cells; 1—a few cells surrounding few bronchioles and vessels; 2—a few cells surrounding multiple bronchioles and vessels, a few clusters of cells scattered in few alveolar spaces; 3—a ring of cells one to two cells deep affecting multiple bronchioles and vessels, a few clusters of cells scattered in multiple alveolar spaces, minimal increased cellularity in interstitium; 4—a ring of cells two to four cells deep affecting multiple bronchioles and vessels, a few clusters of cells scattered in multiple alveolar spaces, mild increased cellularity in interstitium; 5—a ring of cells >4 cells deep affecting most bronchioles and vessels, multiple clusters of cells scattered in most of the alveolar spaces, moderate increased cellularity in interstitium.

### Statistical Analysis

The statistical significance of difference between two groups was analyzed using unpaired Student’s t-test or non-parametric Mann Whitney test. Statistical significance was ascribed to the data when p < 0.05.

## Results

### Calgranulin homodimers induce proinflammatory cytokines production via TLR4

**Fig 1:**
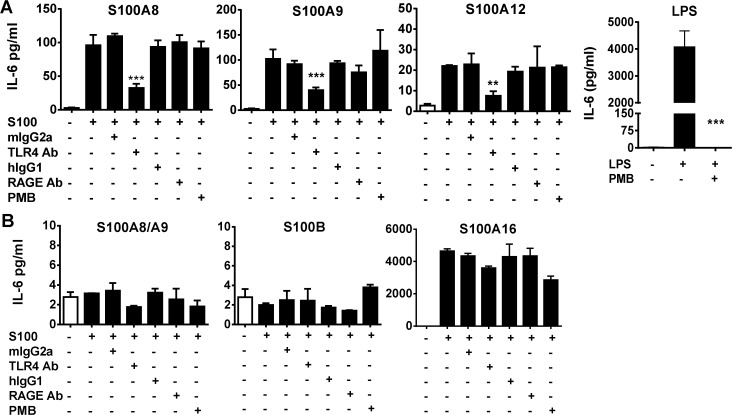
TLR4 but not RAGE is necessary for calgranulin-mediated proinflammatory cytokine induction, but other S100s exhibited variable cytokine responses. Human PBMCs were stimulated with 10 μg/ml of human S100 preparations and levels of IL-6 (and other cytokines) were measured after 16 h in culture. (A) Cells were stimulated with low endotoxin preparations of calgranulins (S100A8, S100A9 or S100A12) or LPS and were treated with either nil, control Ab, anti-hTLR4, anti-RAGE Abs or Polymyxin B (PMB). (B) Cells were stimulated with S100A1, S100A16, S100B and S100A8/A9 and were similarly treated with nil, control Ab, anti-hTLR4 Ab, anti-RAGE Ab, or Polymyxin B. Representative data from one of three experiments is shown. Individual experiments were performed in triplicate and mean ± SD are given. Unpaired T-tests was used to determine if the test antibody differed significantly from their respective control Abs following stimulation, and if the polymyxin treatment differed from no treatment following stimulation (*P<0.05, **P<0.01, ***P<0.001).

Previous data has indicated that S100A12 and S100A8 can induce cytokine responses through RAGE and TLR4 respectively [11,22]. Here we examined a broad range of S100s and assessed their capacity to induce proinflammatory cytokines from PBMC. Microgram quantities of the calgranulins, S100A8, S100A9 and S100A12, induced modest levels of IL-6 from human PBMC at 4 h and 24 h time-points which were significantly inhibited by anti-TLR4 Ab, but not by an anti-RAGE blocking Ab (Fig. 1A). Similarly, anti-TLR4 antibody but not anti-RAGE antibody blocked the induction of modest levels of IFNγ, IL1β, and TNFα (data not shown). The cytokine induction from PBMC was completely dependent on the presence of CD14+ monocytes which is consistent with the expression of TLR4 on these cells. The endotoxin inhibitor Polymyxin B had no effect on cytokine production from these low endotoxin S100 preparations, whereas it completely inhibited LPS induced IL-6 production in human PBMCs (Fig. 1A). In contrast to the calgranulins, up to 30 μg/ml S100A8/A9 heterodimer isolated from neutrophils or recombinant S100B failed to induce any significant cytokine induction, whereas S100A16 induced a strong cytokine response (Fig. 1B). S100A16 induced cytokines were not inhibited by anti-RAGE Ab treatment and only modestly impacted by TLR4 blockade or polymyxin B treatment suggesting that this S100 may drive an inflammatory response through an alternate receptor. Endotoxin contamination did not permit assessment of the cytokine induction from other recombinant S100 preparations.

### Differential effects of anti-RAGE antibody treated on S100-mediated THP-1 cell migration

**Fig 2:**
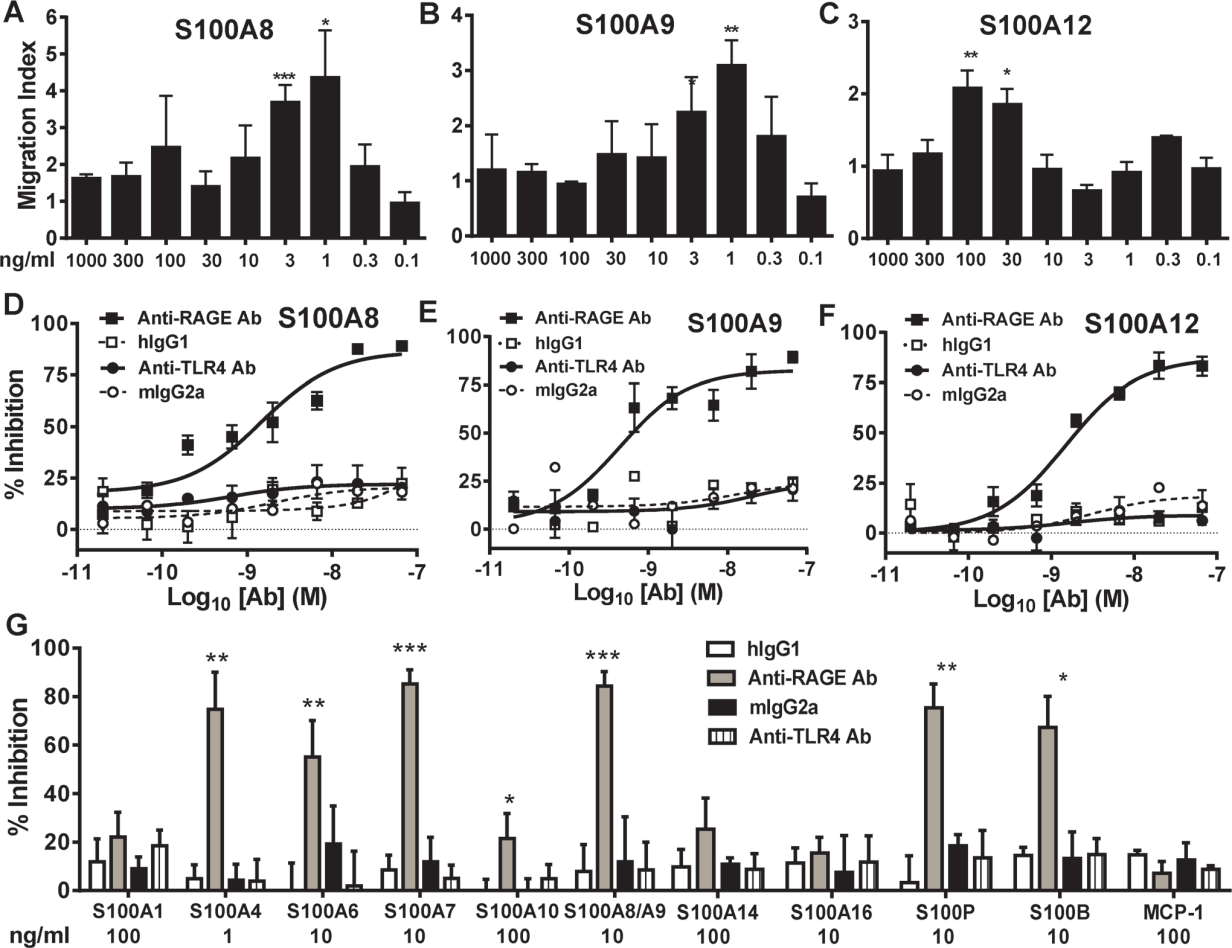
RAGE blockade inhibits calgranulin (hS100A8, hS100A9 or hS100A12) induced THP-1 cell migration but does not impact migration of all S100s. Migration indices for THP-1 cells in response to hS100A8 (A), hS100A9 (B), and hS100A12 (C). Effects of anti-RAGE, anti-hTLR4 Abs and isotype control Abs on hS100A8 (D), hS100A9 (E) and hS100A12 (F) induced THP-1 cell migration. Serial dilution of anti-RAGE, anti-TLR4 or isotype-matched control Ab were incubated with THP-1 cells in the upper wells of the chemotaxis chamber, and optimal amounts of hS100A8 (1 ng/ml), hS100A9 (1 ng/ml) and hS100A12 (100 ng/ml) were added in the lower wells. Percentage inhibition is relative to no Ab treatment. One representative of three independent experiments is shown (mean ±SD of triplicate wells) (G) The effects of a fixed 10 μg/ml dose of anti-RAGE, anti-hTLR4 and control Abs on THP-1 cell migration mediated by other S100s and MCP1. The S100s were used at their optimal concentrations (indicated on graphs). The maximal chemotactic indexes were S100A1 (2.0), S100A4 (2.9), S100A6 (2.9), S100A7 (3.7), S100A8/A9 (3.1), S100A10 (1.9), S100A14 (2.7), S100A16 (2.4), S100P (3.5), S100B (3.5) and MCP1 (9.5). Percentage inhibition is relative to no Ab treatment. Mean inhibition ± SD for one of two independent experiments is shown. Unpaired T-test was used to determine if the anti-RAGE or anti-hTLR4 Ab treatments were significantly different from the isotype control Ab (*P<0.05, **P<0.01, ***P<0.001).

Several S100s have been shown to have chemotactic activities, and RAGE has been implicated in mediating the migration of S100B, S100A4, S100A12 and S100A7, whereas S100A15 appears to be mediated by an as yet unidentified Gi protein coupled receptor [11,36–39]. Chemotaxis of THP-1 cells with 13 S100s and the positive control MCP-1 were examined using a 96-well ChemoTx system. Initially, we demonstrated that the calgranulins S100A8, S100A9 and S100A12 all induced THP-1 cell migration with a characteristic bell-shaped response typical of traditional chemokines (Fig. 2A,B,C). This migration was significantly less if the S100 proteins were put in the upper well indicating that the migration was chemotactic, rather than a consequence of increased motility (data not shown). Using the optimal concentration for each S100 we demonstrate that migration of THP1 cells to S100A8, S100A9 and S100A12 was inhibited in a dose-dependent manner with anti-RAGE Ab, but not an anti-TLR4 Ab (Fig. 2D,E,F). Similarly, S100A1, S100A4, S100A6, S100A7, S100A10, S100A8/A9, S100A14, S100A16, S100P, S100B and MCP-1 all induced migration of THP-1 cells. The chemotactic activities of S100A4, S100A7, S100B, S100P and to a lesser extent S100A6 mediated migration of THP1 cells were also dependent on RAGE. As expected the MCP-1 induced THP-1 migration was unaffected by RAGE or TLR4 blockade. Similarly, S100A1, S100A10, S100A14, and S100A16 induced THP-1 migration was also independent of RAGE and TLR4 (Fig. 2G).

### *In vitro* S100A9-induced migration of leukocytes is RAGE dependent

**Fig 3:**
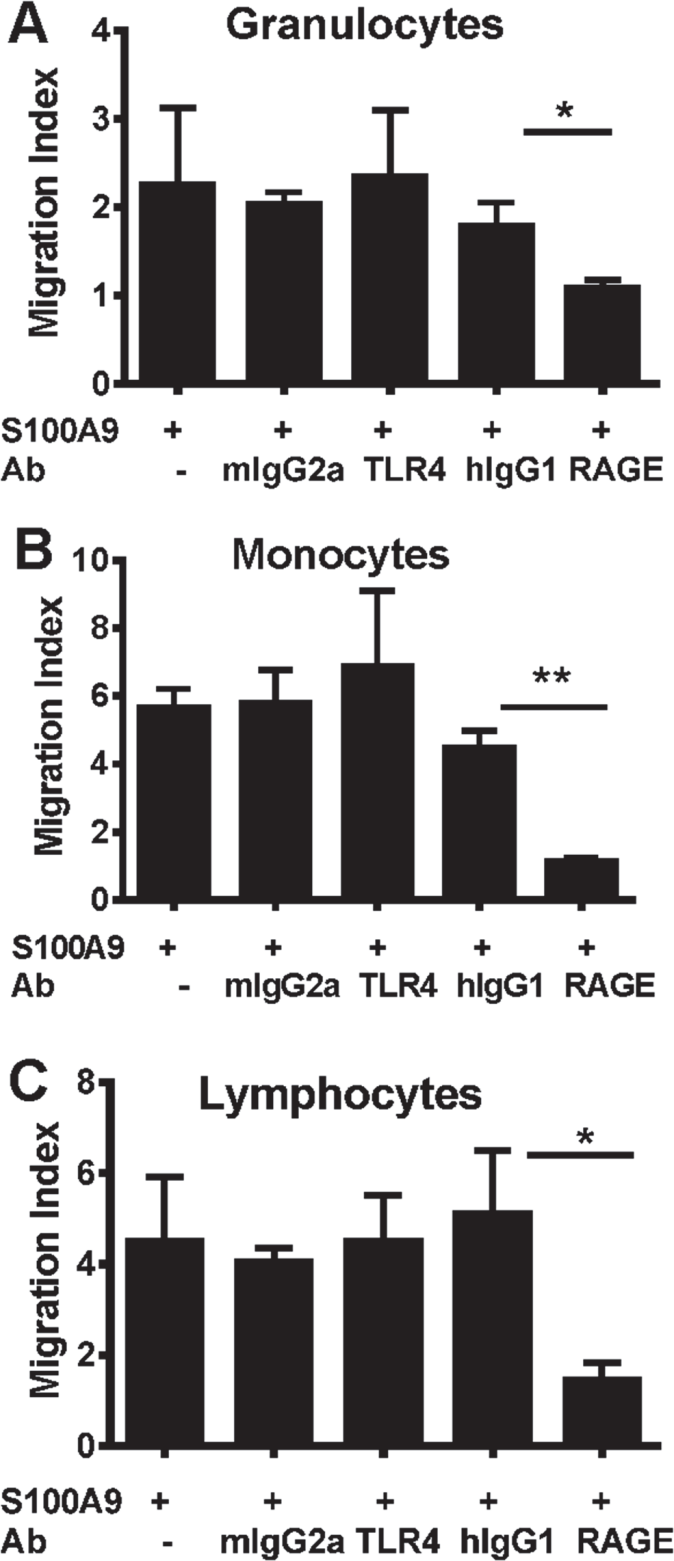
Human S100A9 induced migration of leukocyte populations is inhibited by anti-RAGE Ab but not anti-TLR4 Ab. Chemotactic responses to S100A9 of human leukocyte populations were examined at the optimal concentration (1 ng/ml). The effects of TLR4 and RAGE blockade on S100A9-mediated migration of granulocytes (A), monocytes (B), and lymphocytes (C), with 10 μg/ml of anti-RAGE, anti-hTLR4 Ab and isotype control Abs. Data shown is mean±SD of triplicate samples from a representative of three independent experiments. Unpaired T-test was used to determine if the anti-RAGE or anti-TLR4 treatments were significantly different from the isotype control Ab (*P<0.05, **P<0.01, ***P<0.001).

Since the *in vitro* experiments hitherto have been focused on monocytic THP1 cells, we next examined the migratory effects of S100A9 on various primary cells. These experiments demonstrated that this calgranulin significantly induced the migration of granulocytes, lymphocytes and monocytes isolated from healthy human volunteers. As observed with the THP1 cells, the migration of granulocytes, monocytes and lymphocytes induced by S100A9 required RAGE but not TLR4 (Fig. 3).

### S100A9 induced THP-1 cell migration requires signaling by MEK/ERK, nuclear factor-κB and PI3K but not p38

**Fig 4:**
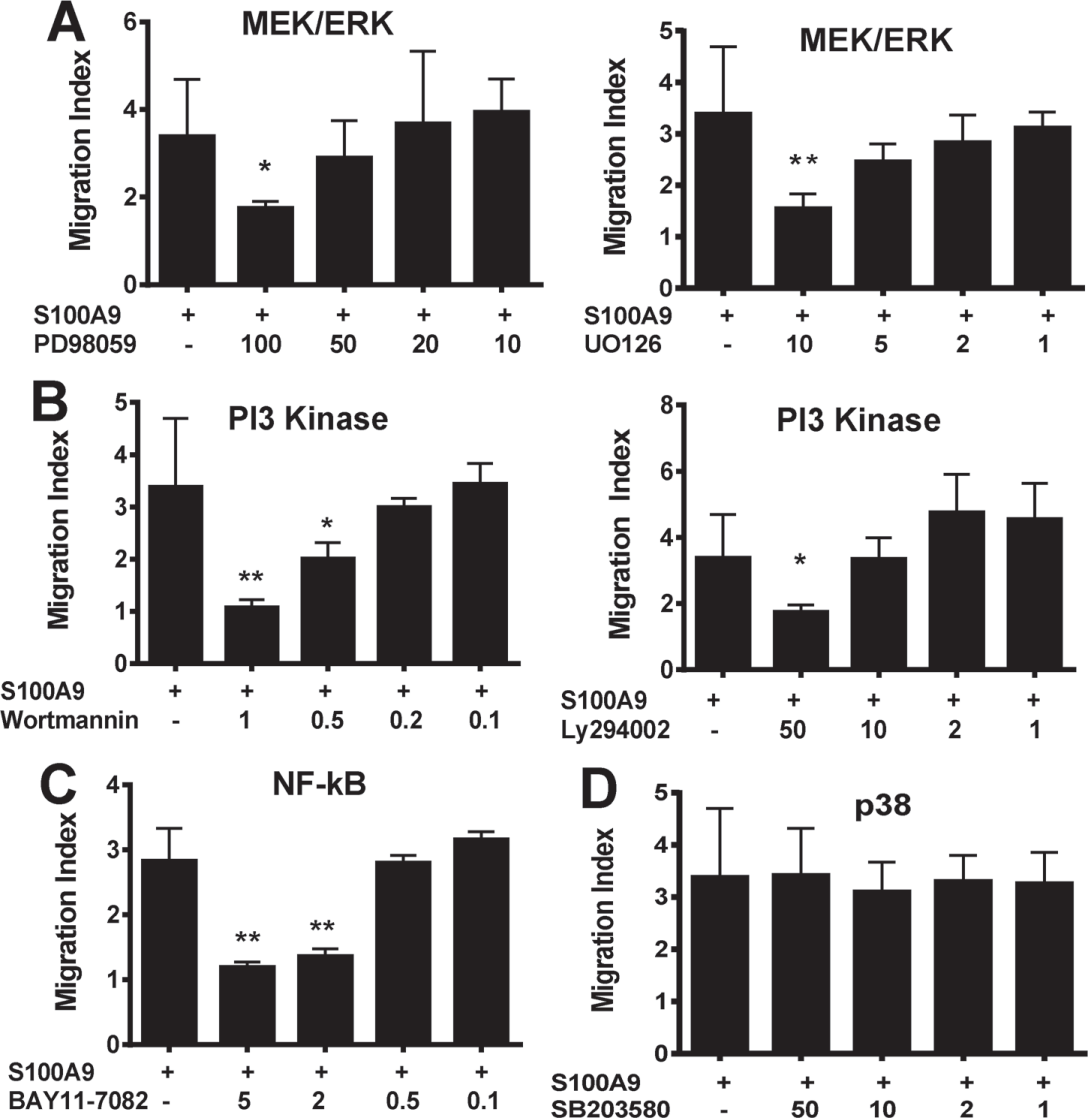
Human S100A9 induced THP-1 cell migration requires MEK/ERK and PI3K but not p38. Chemotactic response of THP-1 towards hS100A9 (1 ng/ml) in the absence or presence of specific inhibitors targeting the MEK/ERK pathway (A), the PI3 kinase pathway (B), the NF-κB pathway (C), and the p38 MAPK pathway (D). The inhibitor concentrations (μM) are indicated. Data shown is mean±SD of triplicate samples from a representative of three independent experiments.

Previous data has indicated that engagement of RAGE by its ligands can trigger the activation of a broad range of signaling pathways including Ras/MEK/ERK 1/2 dependent NFkB activation, CDC42/Rac/MAPKK dependent p38 activation, SAPK/Jnk MAP kinases dependent AP-1 activation as well as the phosphatidylinositol 3-kinase (PI3K)/AKT activation [37,40–42]. To ascertain which signaling pathway(s) are required for S100A9-RAGE triggered cell migration, we examined the effects of inhibitors targeting the MEK/ERK, NF-κB, p38 and PI3 kinase pathways on cell migration. The MEK/ERK pathway inhibitors (PD 98059 and UO126), PI3 kinase inhibitors (Wortmannin and Ly294002), and NF-κB inhibitor BAY11-7082 inhibited S100A9 induced THP-1 cell migration in dose-dependent manner (Fig. 4A-C), whereas p38 MAPK inhibitor SB203580 failed to block S100A9 induced THP-1 cell migration (Fig. 4D). None of the inhibitors induced any significant cytotoxicity at their highest concentrations (data not shown). Consistent with these data, S100A9 induced modest by significant increases in ERK1/2 and AKT phosphorylation, whereas total ERK1/2 and AKT levels remained unchanged (S1 Fig.). Notably, increased phosphorylation of ERK1/2 and AKT was associated with the higher levels of S100A9 necessary to induce cytokines. Thus, S100A9 induced THP-1 cell migration requires MEK/ERK, NF-κB and PI3K activity, but not p38 MAPK.

### Effects of Anti-RAGE and Anti-TLR4 Abs on murine S100A9 induced RAW cell migration and proinflammatory cytokine induction

**Fig 5:**
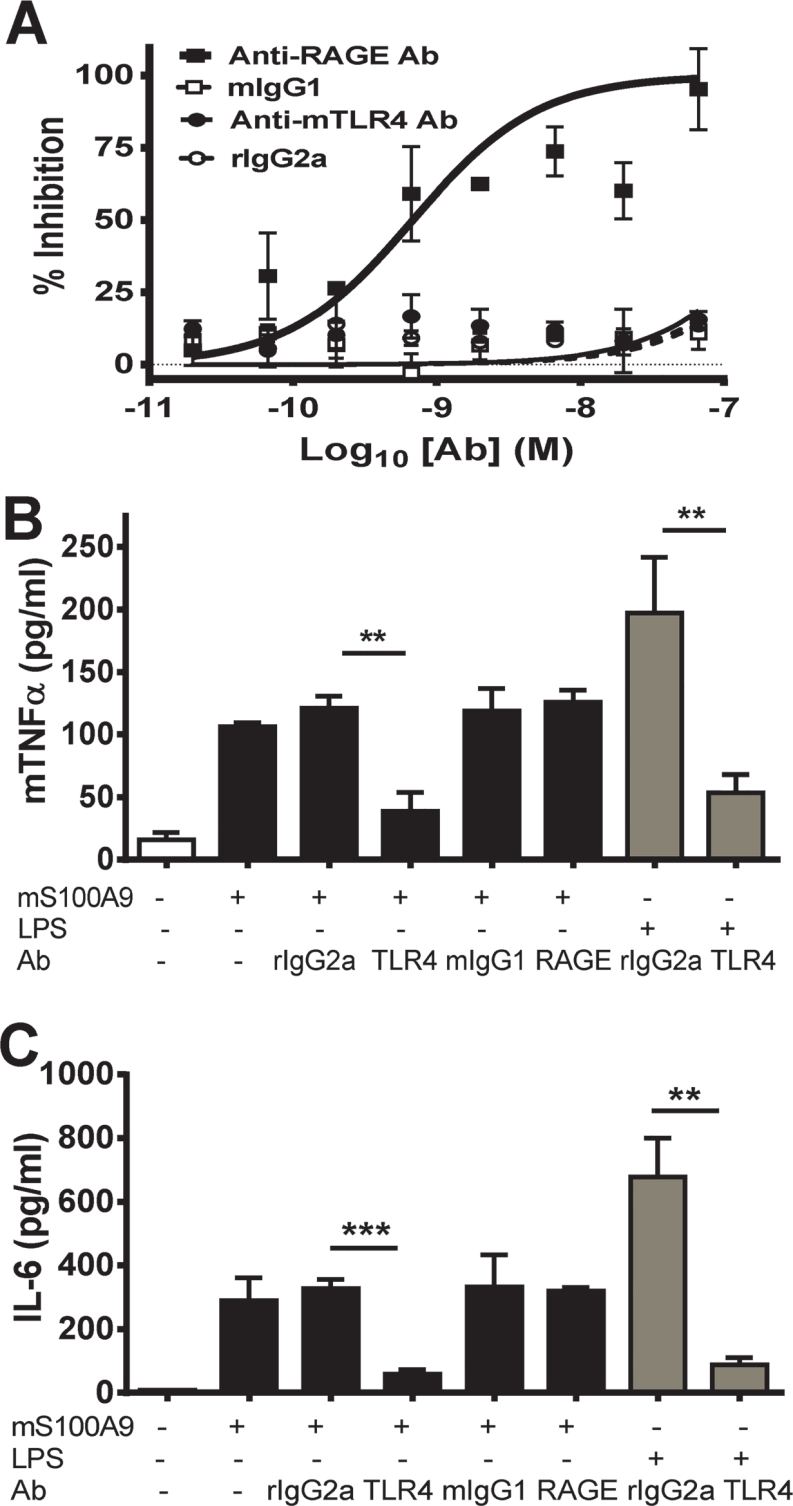
Effect of RAGE and TLR4 blockade on murine S100A9 induced RAW cell migration and proinflammatory cytokine induction. (A) Murine S100A9 (1 ng/ml) was used to induce the migration of RAW cells treated with dose titrations of anti-RAGE, anti-murine TLR4 Ab or isotype control Abs. Percentage inhibition is relative to no Ab treatment. Murine S100A9 (10 μg/ml) or LPS was used to induce TNFα (B) and IL-6 (C) from RAW cells with or without anti-RAGE, anti-mTLR4/MD2 or control Abs. Unpaired T-test was used to determine if the anti-RAGE or anti-mTLR4/MD2 treatments were significantly different from the control Abs for each treatment group (*P<0.05, **P<0.01, ***P<0.001). Data shown is mean±SDEM of triplicate samples from a representative of three independent experiments.

In preparation for *in vivo* studies, we next investigated whether the activities of S100A9 were similar between human and mouse. To this end, we generated small amounts of endotoxin-free mammalian expressed recombinant murine S100A9. At low concentration (1 ng/ml) mS100A9 induced significant migration of RAW cells, a murine macrophage cell line. This response was completely abrogated by an anti-RAGE Ab whereas the anti-mTLR4/MD2 Ab had no effect (Fig. 5A). At a higher concentration (10 μg/ml), murine S100A9 induced the production of the TNFα and IL-6 (Fig. 5 B, C). Induction of cytokines was significantly inhibited by anti-mTLR4/MD2 Ab, but not by the anti-RAGE Ab. The activity of the anti-mTLR4/MD2 Ab was demonstrated by its capacity to inhibit LPS induced cytokine induction. Overall, these data suggest that human and murine S100A9 trigger pro-inflammatory responses *in vitro* through the same receptors and they appear to be functionally equivalent.

### mS100A9-mediated lung inflammation is RAGE-independent

**Fig 6:**
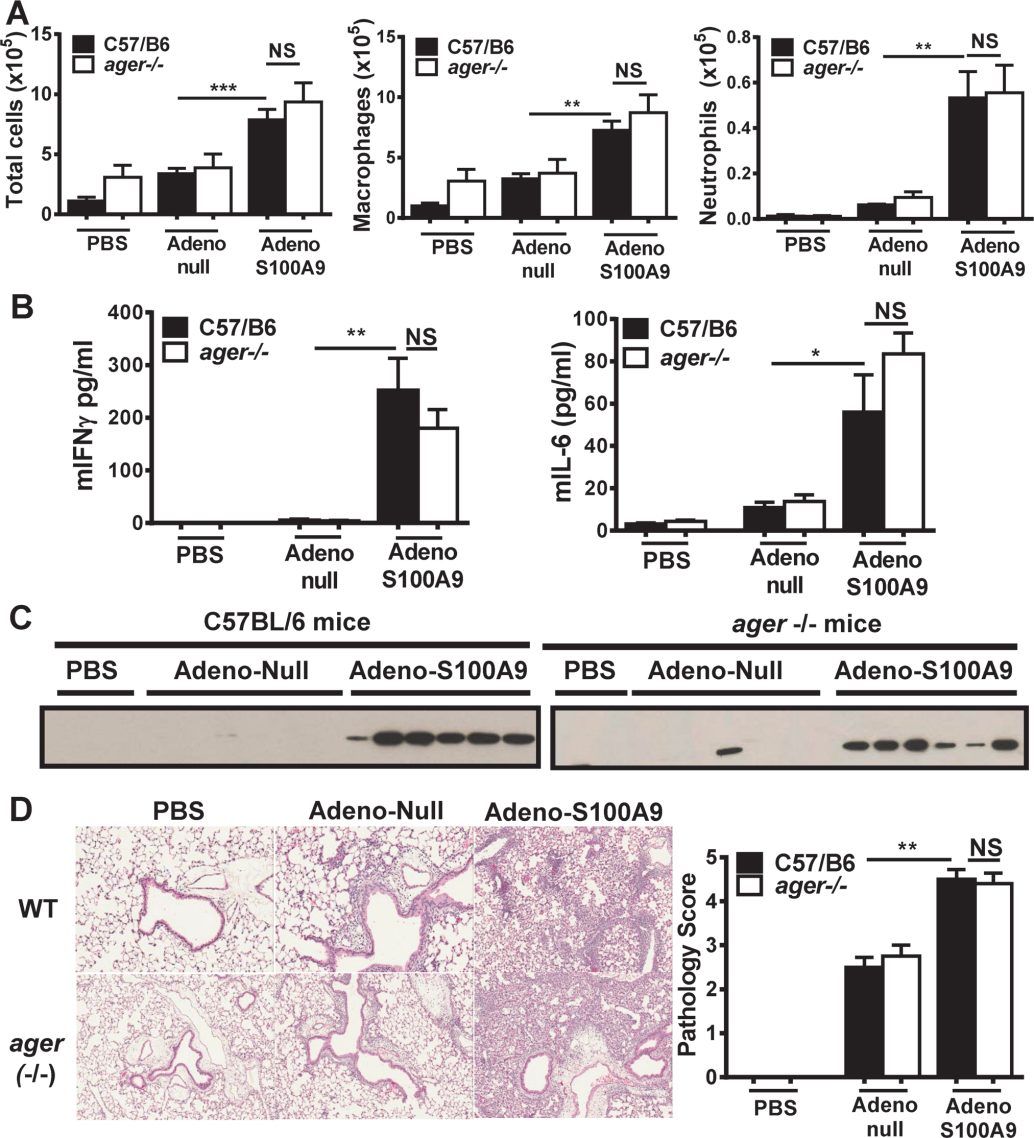
Murine S100A9 induced lung inflammation is RAGE-independent. Wild type C57Bl/6 or C57Bl/6 *ager*-/- mice were challenged intranasally with PBS, or adeno-null or adeno-murine S100A9, after 8 days, mice were sacrificed and BAL fluids or lung tissue were collected for analysis. (A) Cells were obtained from the BAL fluids using cytospins, stained with diff-quik and the total cell counts, neutrophil counts and macrophage counts were recorded. (B) mIFNγ and mIL-6 proteins levels found in BAL fluids of wild type and *ager*-/- mice. (C) Western blot analysis of murine S100A9 levels in BAL fluids. (D) H&E staining of paraffin fixed lung tissue (left panel), and pathology scores (right panel). Non-parametric Mann-Whitney test was used to determine statistical difference between two groups.

It was previously shown that blockade of S100A8 and S100A9 with polyclonal antibodies prevented phagocyte migration into alveolar space in mice challenged with LPS or *Streptococcus pnuemoniae* [20]. While S100A8 and S100A9 were clearly contributing to the inflammation observed in these models the receptor(s) responsible were not delineated. To directly assess the inflammatory role of S100A9 *in vivo*, we generated an adenovirus encoding murine S100A9 which was intranasally administered to naive mice. The use of an adenoviral system was used to avoid the possibility of trace amounts of endotoxin associated with recombinant protein influencing results. At early time-points (24 h and 72 h), there was a weak airway inflammation associated with the adenoviral challenge. At Day 5, increased cellular recruitment to the airways coincided with expression of mS100A9 in the adeno-mS100A9 challenged mice. By 8 days post infection, the cellular infiltrates in the BAL fluids of the C57BL/6 wild-type mice were significantly increased in the adeno-mS100A9 treated group compared to the adeno-null group (Fig. 6A). The cellular infiltrates were primarily composed of macrophages, with small numbers of neutrophils and lymphocytes. Interestingly, there was no significant differences in the BAL fluid inflammatory infiltrate between the wild-type and *ager* -/- (RAGE-deficient) mice following adeno-mS100A9 administration (Fig. 6A). We also observed significantly increased levels of IFNγ and IL-6 in the BAL fluids of adeno-mS100A9 infected mice compared to the adeno-null controls, but again there were no differences between the mS100A9-adenovirus infected wild-type and *ager* ^-/-^ mice (Fig. 6B). TNFα and IL1β were also examined but levels were below detection. To ensure that the increased inflammatory responses with the adeno-S100A9 we observed in the BAL fluids could be attributed to S100A9, we assayed for the presence of mS100A9 in the BAL fluid. Western blot analysis of the BAL fluids revealed that murine S100A9 was almost exclusively detected in the adeno-S100A9 challenged groups at Day 8 (Fig. 6C). There was no apparent difference between the wild-type and *ager*-/- mice. Using recombinant mS100A9 as a reference, the levels of mS100A9 recovered from the BAL fluids was estimated to be ∼1–10 μg. In the lung tissue, there was a mononuclear cell infiltrate (predominantly macrophages) surrounding multiple bronchioles and vessels, with a mild increase in cellularity in the interstitium and air spaces in the adeno-null treated groups whereas inflammatory cells were either absent or rare in the PBS controls (Fig. 6D). The adeno-mS100A9 treated mice had a significantly more severe peribronchiolar, perivascular, and interstitial inflammatory cell infiltration, consisting predominantly of macrophages and lymphocytes, which occasionally extended into the alveolar spaces (Fig. 6D). There were no apparent differences in the histopathological assessments of the wild-type and RAGE-deficient animals for the adeno-mS100A9 treated groups (Fig. 6D). Besides using RAGE-deficient mice, the effect of RAGE blockade was also examined. Administration of anti-RAGE Ab (15 mg/kg i.p) on Day 4 and Day 7 post adeno-S100A9 infection of C57Bl/6 mice provided exposure to the lung, but had no impact on the cellular inflammation or the cytokines at Day 8 (data not shown). These results demonstrate that RAGE does not appear to play a role in S100A9-mediated cytokine induction and inflammation in this *in vivo* model.

### Defective TLR4-signaling reduced S100A9-mediated cytokine induction but had no significant impact on cellular recruitment to the lung tissue and airways

**Fig 7:**
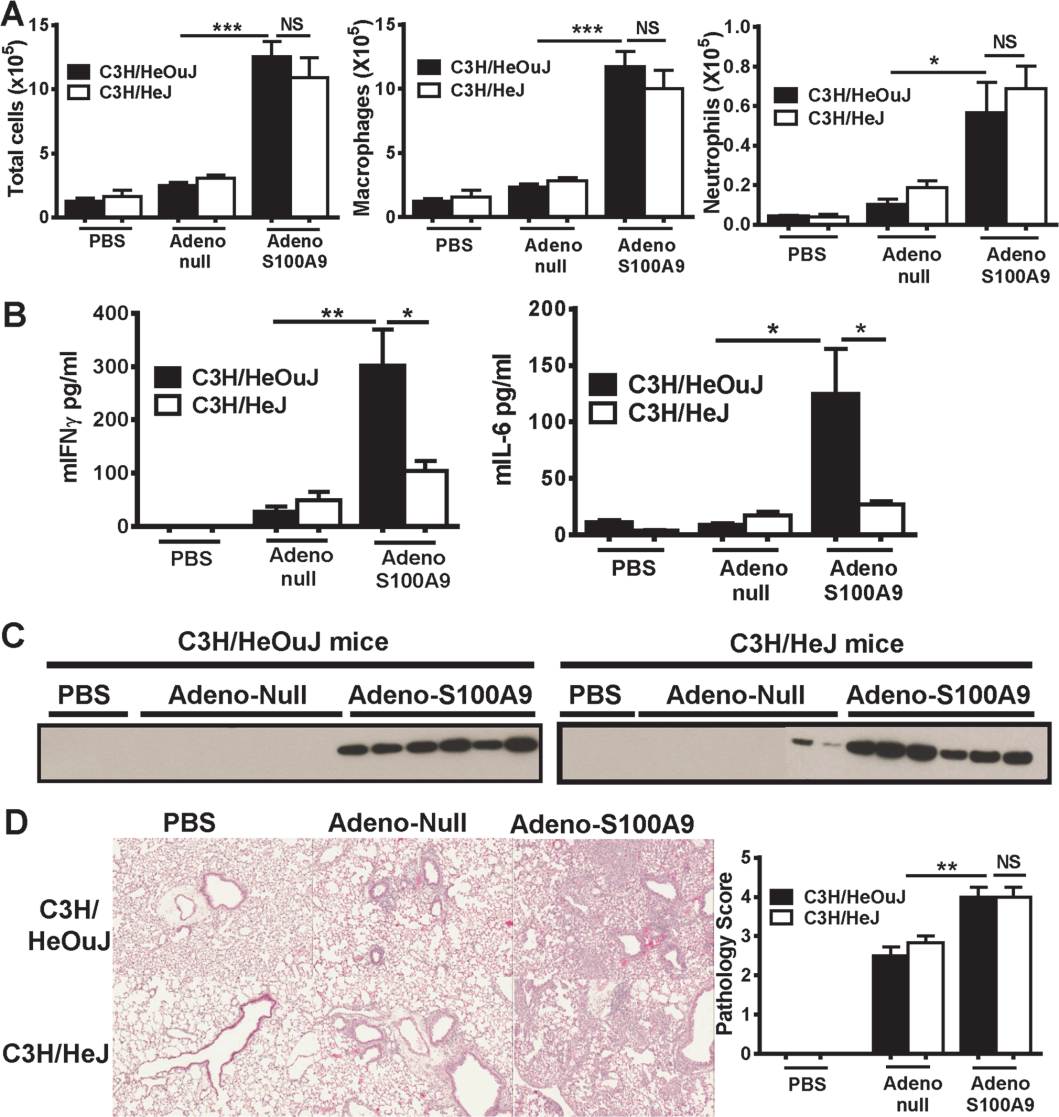
Murine S100A9 induced inflammatory infiltrates are TLR4-independent. Adeno-murine S100A9, Adeno-null or PBS was administered intranasally into wild-type (C3H/HeOuj) and TLR4-defective (C3H/HeJ) mice. After 10 days, mice were euthanized and BAL fluids or lung tissue was collected for analysis. (A) Cells were obtained from the BAL fluids using cytospins, stained with diff-quik and the total cell counts, neutrophil counts and macrophage counts were recorded. (B) mIFNγ and mIL-6 expression in BAL fluid in wild type and TLR4 defective mice. (C) Western blot analyses of mS100A9 expression in BAL fluid in wild type and TLR4 defective mice. (D) H&E staining of paraffin fixed lung tissue (left panel), and pathology scores (right panel). Non-parametric Mann-Whitney test was used to determine statistical difference between two groups.

Since the levels of murine S100A9 recovered from the lung in the BAL fluids were sufficient to trigger TLR4 *in vitro*, we then investigated whether there was a role for TLR4 in adeno-mS100A9 induced cellular infiltration and proinflammatory cytokine induction. TLR4-defective (C3H/HeJ) and wild-type (C3H/HeOuJ) mice were intranasally infected with adeno-mS100A9 or adeno null virus and examined 10 days post infection (as the kinetics of the C3H/HeOuJ response were slightly delayed compared with C57Bl/6 mice). Analysis of the BAL fluid samples revealed the adeno-mS100A9 induced a significant predominately macrophage cell infiltration in the airways compared to the adeno-null controls. There were no differences between the wild-type (C3H/HeOuJ) and TLR4-defective (C3H/HeJ) mice (Fig. 7A). Adeno-mS100A9 induced a robust proinflammatory cytokine response (IL-6 and IFNγ) in the wild-type (C3H/HeOuJ) mice compared to the control adeno-null groups (Fig. 7B). This S100A9-mediated cytokine response was markedly ablated in TLR4-defective mice (Fig. 7B), which is consistent with the *in vitro* data which demonstrated that anti-TLR4 antibodies could inhibit S100A9-mediated proinflammatory cytokine induction (Figs. 1A,B and 5B,C). It is notable that these significant reductions in the BAL cytokines had no impact on the cellular infiltrates in the airways between the wild-type and TLR4-defective mice (Fig. 7A,B). Similar levels of S100A9 levels were detected in the BAL fluids of wild-type (C3H/HeOuJ) and TLR4-defective (C3H/HeJ) adeno-mS100A9 infected mice (Fig. 7C). In the lung tissue there was a comparable, mild perivascular mononuclear inflammatory infiltrate in the adeno-null treated wild-type (C3H/HeOuJ) and TLR4-defective (C3H/HeJ) mice relative to the PBS treated mice. Both the wild-type and TLR4-defective adeno-S100A9 challenged mice had a much more severe inflammatory infiltrate with marked increases in the cellularity surrounding vessels and bronchioles, and extensive infiltration of the interstitium and to a lesser extent in the alveolar spaces (Fig. 7D). There was no apparent qualitative difference in the lung pathology exhibited by the adeno-S100A9 treated wild-type and TLR4-defective mice (Fig. 7D). Overall these data indicate that neither RAGE nor TLR4 is required for driving the mS100A9-mediated cellular inflammation in the lung.

## Discussion

In the lung, increased levels of S100A8, S100A9 and S100A12 have been found in asthma, cystic fibrosis, chronic obstructive pulmonary disease, idiopathic pulmonary fibrosis, acute respiratory distress syndrome and ventilator associated lung injury [10,31–33,43–46]. The relative contributions of S100A8, S100A9, S100A8/A9 and S100A12, and the receptors that mediate their functions under physiological conditions remain unclear. In different contexts, the putative receptors TLR4 and RAGE have also been shown to mediate acute and chronic lung inflammation [47,48]. This would infer that targeting S100 proteins or their receptors may provide a viable strategy for the treatment of inflammatory lung disorders, however, the *in vivo* studies conducted to date have not adequately linked the S100A8, S100A9 or S100A8/A9 with their putative receptors RAGE or TLR4, so a better understanding of the biology is necessary to identify the most appropriate therapeutic approach.

Here we report the following significant findings; i) *in vitro*, S100s can induce chemotaxis and in most cases this can be inhibited by anti-RAGE Abs, whereas S100A8, S100A9 and S100A12 induced modest levels of cytokines from monocytes that were inhibited by anti-TLR4 treatment, ii) cell migration and cytokines induced by human and murine S100A9 was inhibited *in vitro* by anti-RAGE and anti-TLR4 Abs respectively, iii) S100A9 homodimers generated *in vivo* following adenoviral delivery are sufficient to induce a robust inflammatory response in the lung, and iv) contrary to the *in vitro* data, the robust murine S100A9-mediated cellular inflammation induced in this simple model was independent of both RAGE and TLR4.

Intranasal delivery of adenoviral mS100A9 to the lungs of mice was sufficient to induce a robust airway inflammation. The peak of the S100A9-dependent inflammation was 8–10 days post-infection and this timing coincided with the highest levels of mS100A9 recovered from the BAL fluid (∼1–10 μg). It is clear that the levels of S100A8 and S100A9 are increased in a range of different lung disorders, but to date, no standardized methods for the collection, detection or reporting of S100A8, S100A9 or S100A8/A9 levels from clinical samples from the lung have been established to allow direct comparisons to be made. However, approximately, 13 μg /ml of S100A8/A9 was recently detected in the lung lavage of patients with acute lung injury, and ∼ 1 to 5 μg /ml of S100A8/A9 were recovered from mice with ventilator induced lung injury in the presence and absence of LPS [46]. The levels of mS100A9 we detect in our model are consistent with these amounts, and those necessary to trigger cytokine induction and transendothelial migration *in vitro* [30]. Therefore, we predict that the mS100A9 levels generated in our murine model are likely to be physiologically relevant. Most surprisingly, despite the capacity to induce TLR4-mediated cytokine induction and RAGE-dependent migration *in vitro*, our data demonstrate that S100A9-mediated airway cell recruitment/inflammation is independent of both these receptors.

Our *in vitro* studies demonstrated that S100A1, S100A4, S100A6, S100A7, S100A8, S100A9, S100A8/A9, S100A10, S100A14, S100A16, S100B and S100P all have chemotactic activity towards THP1 cells although the potencies varied. An anti-RAGE Ab, that targets the V-C1 domains of RAGE which are responsible for ligand binding, blocked the majority of migration induced by S100A4, S100A7, S100A8, S100A8/A9, S100A9, S100A12, S100P and S100B. This dataset is consistent with a series of previous studies that have shown chemotactic activity for S100A4, S100A7, S100A8, S100A8/A9, S100A12, S100A15 and S100B towards different cell types [11,18,19,36,38,39], and that the migration of S100A4, S100A7, S100A12 and S100B is RAGE-dependent [36–39]. Our results have extended the list of S100s that can induce migration *in vitro*, and those inhibitable by RAGE blockade. Interestingly we also demonstrate that the RAGE-dependent S100A9 mediated migration was not restricted to neutrophils and monocytes, but was also evident for lymphocytes, particularly T cells and NK cells. The significance of this unexpected finding warrants further exploration.

Our analysis of the signaling pathways necessary for S100A9-induced migration of THP1 cells clearly identified a requirement for the MEK/ERK, PI3K and NF-κB pathways, but not for p38. This is consistent with recent data that has shown S100A9 promotes lung fibroblast cell activation through RAGE mediated ERK1/2, MAPK and NF-κB signaling [49], and a detailed report on the RAGE-dependent signaling induced by S100B on microglia cells which required the recruitment of diaphanous-1 and activation of Src kinase, Ras, PI3K, MEK/ERK1/2, RhoA/ROCK, Rac1/JNK/AP-1, Rac1/NFkB, and, to a lesser extent, p38 MAPK [38].

It is noteworthy that the S100A9 concentrations appropriate for optimal cell migration were insufficient to induce cytokine induction, and anti-TLR4 antibody treatment had no impact on cell migration in these *in vitro* assays. Moreover, the THP1 cells were not chemotactic to LPS or high concentrations of S100A9 *in vitro* either, so TLR4 clearly does not mediate a direct chemotactic response. Although aspects of the RAGE and TLR4 signaling pathways are similar, the differential cellular responses mediated by RAGE and TLR4 likely reflect differences in the binding of the S100A9 homodimer to the respective receptors, mechanisms or thresholds of receptor activation, the magnitude of down-stream signaling. Indeed, the concentrations of S100A9 required to induce cytokines were associated with much greater ERK1/2 and AKT phosphorylation. Moreover, S100A9 induced cytokine induction is associated with greater NF-κB activation than LPS stimulation and TLR4 internalization [24].

The partial inhibition of S100A6 with the anti-RAGE Ab is consistent with binding studies that indicate that it can also bind both the V-C1 and C2 domains of RAGE [40]. Migration of S100A1, S100A10, S100A14 and S100A16 were not inhibited by RAGE blockade. It is plausible that these S100A1 may bind an alternate RAGE binding site not blocked by the antibody since it has been shown to bind the V-domain of RAGE and induce neurite outgrowth [14,37]. However, a thorough analysis of the interactions between RAGE and S100s did not identify interactions between RAGE and S100A10, S100A14 or S100A16 [14]. These data imply that several S100s may induce migration independent of RAGE and an as yet unknown receptor may be necessary, as has been previously proposed for S100A15 [36].

Interestingly, despite a significant literature implicating RAGE in the migration of S100s, and the impact of RAGE blockade on S100-mediated migration described herein, our *in vivo* data demonstrated that murine S100A9-mediated airway inflammation is independent of RAGE. Previously it has been shown that anti-S100A8 and anti-S100A9 antibodies blocked infiltration of phagocytes into the alveolar space following intranasal challenge with *Streptococcus pnuemoniae* [20] and the migration of macrophages and tumor cells to the lung [50], but the receptors responsible were not determined. Furthermore, RAGE-deficient mice had reduced infiltration into the lung tissue and airspace following challenge with *Streptococcus pnuemoniae*, Influenza A virus or Respiratory syncytial virus [34,48,51]. In these cases the complex mechanism by which RAGE mediates lung inflammation was not resolved. On the basis of our *in vitro* data and these *in vivo* studies, we predicted that S100A9-mediated inflammation induced in our airway model would be mediated through RAGE. However, our data with RAGE-deficient mice and RAGE blockade with a ligand-blocking antibody in wild-type mice clearly demonstrate that RAGE appears to be redundant in S100A9-induced lung and airway inflammation. Most *in vivo* studies have examined S100 induced responses in acute settings were the inflammation is predominately neutrophilic, whereas our model demonstrated that S100A9 can drive a delayed predominately macrophage inflammatory response. It is plausible that RAGE may differentially mediate recruitment of neutrophils and macrophages in response to S100A9, however, the S100A8- and S100A9-dependent neutrophil inflammation induced by monosodium uric acid crystals in a murine air-pouch model [18,19], was also unaffected by RAGE deficiency (data not shown). S100s have been regularly reported as potent chemoattractants since low concentrations can induce cellular migration *in vitro*. However, it has been postulated that the concentrations of calgranulins encountered at inflammatory sites can often be several orders of magnitude higher which could preclude their chemotactic activity [52]. This perspective may well have some validity, but it was recently shown that higher levels of S100A9 akin to those found *in vivo* during inflammatory responses were necessary to trigger transendothelial migration *in vitro* [30]. We cannot rule out the possibility that RAGE-dependent effects were subtle and not detected in our experimental system, or other factors may be necessary to promote RAGE-dependent responses *in vivo*. It is known that RAGE ligands and other inflammatory mediators can upregulate the expression of RAGE which could promote RAGE-dependent effects [53]. This possibility could also be complicated by the promiscuous nature of RAGE and its capacity to engage and respond to other DAMPs such as HMGB1 and nucleic acids [3,54], and the presence of both soluble and membrane forms that may play opposing roles *in vivo* [34]. An explanation that befits the data in our simple experimental model is that RAGE does not play a direct physiological role in S100A9-mediated inflammation. Whether or not this extends to S100A8/A9 heterodimers, S100A8 and potentially other S100s was beyond the scope of this study, but it is plausible, and warrants further investigation.

We considered alternate receptors that could mediate S100-mediated inflammation and the possibility that TLR4-mediated cytokine induction may drive the lung inflammation. *In vitro*, we showed that S100A8, S100A9 and S100A12 induce similar modest levels of TNFα, IL-6, IL-1β and IFNγ in a TLR4-dependent manner, and the response could not be attributed to endotoxin contamination. Considering that both S100A8 and S100A9 induced cytokines, it is perhaps surprising that the S100A8/A9 heterodimer isolated directly from neutrophils failed to induce cytokines. A previous report indicated that S100A8 and the S100A8/A9 heterodimer, but not S100A9 can induce TLR4-dependent cytokine induction [22]. It is plausible that alternate methods to constitute S100A8/A9 heterodimers or extract them from neutrophils may account for the differences, but the capacity of S100A8/A9 to induce cell migration in this study was intact which indicates that the molecule was functional. Similarly, in our hands up to 30 μg/ml of S100B used herein also failed to induce cytokines from mononuclear cells, although low concentrations were sufficient to induce RAGE-dependent migration. In contrast, high doses of S100B have been shown to mediate RAGE-dependent induction of COX-2 and cytokines from microglial cells [55], which indicates that RAGE can promote S100-mediated cytokine induction under some circumstances and the cell type or microenvironment may influence cellular responsiveness. The discrepancy in the induction of cytokines with S100A9 between our data and an aforementioned study [22] is not obviously apparent, but could relate to the stability of the purified protein. However, our data is supported by a subsequent study that showed S100A9 binds MD2/TLR4 [29], and a recent study that S100A9 can induce cytokines via TLR4 [24]. Moreover, we did show a similar response from mammalian expressed endotoxin-free mS100A9 *in vitro*, and by adenovirus-mS100A9 infection *in vivo*, which resulted in the induction of IL-6 and IFNγ in a TLR4-dependent fashion. Surprisingly, despite the impact of TLR4 defective signaling on the cytokine production in the BAL fluid, the S100A9-induced cellular infiltration into the airways could not be attributed to TLR4 in this model. This likely relates to the relatively modest capacity of S100A9 to induce proinflammatory cytokines. Whether S100A9 promotes a stronger TLR4-dependent response in the presence of other proinflammatory mediators to drive pathological inflammatory responses is plausible. TLR4 null mice were not examined in this study so a role for TLR4 independent of its signaling, although unlikely, was not formally ruled out. Previous *in vivo* studies that implicated TLR4 in S100A8 and S100A8/A9 mediated inflammation have done so largely in the context of endotoxemia, where TLR4 would be readily activated [22,46]. Interestingly, in the absence of LPS or mechanical injury, intranasal delivery of recombinant S100A8 or S100A8/A9 induced a modest acute neutrophil accumulation without a notable increase in cytokines or chemokines [46], which would also indicate that S100A8 and S100A8/A9 can induce inflammation independent of TLR4.

Recent work also indicates that activation of the inflammosome may promote S100A8 and S100A9 mediated cytokine induction [23]. Activation of multiple signaling pathways may well heighten the activation status of the cell and consequently contribute to a more profound inflammatory response. An inflammatory environment may also promote a S100A9-dependent response by increasing resistance to proteolytic cleavage and the stability of S100A9 homodimers [15]. Oxidative modifications of S100A8 and S100A9, akin to those described for HMGB1, have been described that influence inflammatory responses [56,57], so it is possible that these or other post-translational modifications may influence the stability and biological activities of S100A9 *in vivo*. We did not examine this eventually in the context of our adenoviral model, but it is possible that infection of the lung epithelial with the adenovirus may generate a more active and resilient homodimer than recombinant material generated from cell lines. In any case, the adenoviral system we used was able to induce a robust S100A9-dependent cellular inflammation in the lung over several days independent of both RAGE and TLR4. The mechanism underlying this response requires further investigation.

Previously, it has been shown that S100A9 and S100A8/A9 bind to sulfated glycosaminoglycans and carboxylated glycans on endothelial cells through interactions with S100A9 rather than S100A8 [58,59]. Complexes of S100A8/A9 and arachidonic acid also bind to the scavenger receptor CD36 which can also be expressed on endothelial cells [60]. Interactions of this sort could establish an attractive S100 substratum for neutrophils and monocyte attachment, activation and transendothelial migration and thereby mediate tissue inflammation. It remains to be determined if the S100-mediated inflammation is mediated through a diverse set of interactions with altered protein or sugar moieties, or through interactions with specific receptors.

In conclusion, we have shown that S100A9, an established biomarker for chronic inflammatory diseases, is sufficient to induce a robust inflammatory response *in vivo*, and therefore, this DAMP or its respective receptors represent potential targets for therapeutic intervention. Since the inflammatory response is independent of its recognized receptors, RAGE and TLR4, it is apparent that while S100A9 remains a *bone fide* target, there remains much to be learnt about the molecular interactions and mechanisms by which calgranulins modulate inflammatory processes *in vivo*.

## Supporting Information

S1 Fig Human S100A9 induces activation of MEK/ERK and PI3K pathways in THP-1 cells. THP-1 cells were treated with S100A9 at indicated concentration for 30 mins, cell lysates were collected, and Phospho ERK1/2 and total ERK1/2 were measured by Phospho(Thr202/Tyr204; Thr185/Tyr187)/total ERK1/2 assay whole cell lysate kit (A), PhosphoAKT and total AKT were measured by Phospho(Ser473) /Total AKT assay whole cell lysate kit (B). Data shown is mean±SD from three independent experiments. *P<0.05, **P<0.01, ***P<0.001 versus control cultures.
(TIF)
