# Supplementary figures and images for: S100A9 Induced Inflammatory Responses Are Mediated by Distinct Damage Associated Molecular Patterns (DAMP) Receptors In Vitro and In Vivo
Source: PLoS One. 2015 Feb 23;10(2):e0115828. doi: 10.1371/journal.pone.0115828 (PMC4338059; doi:10.1371/journal.pone.0115828)

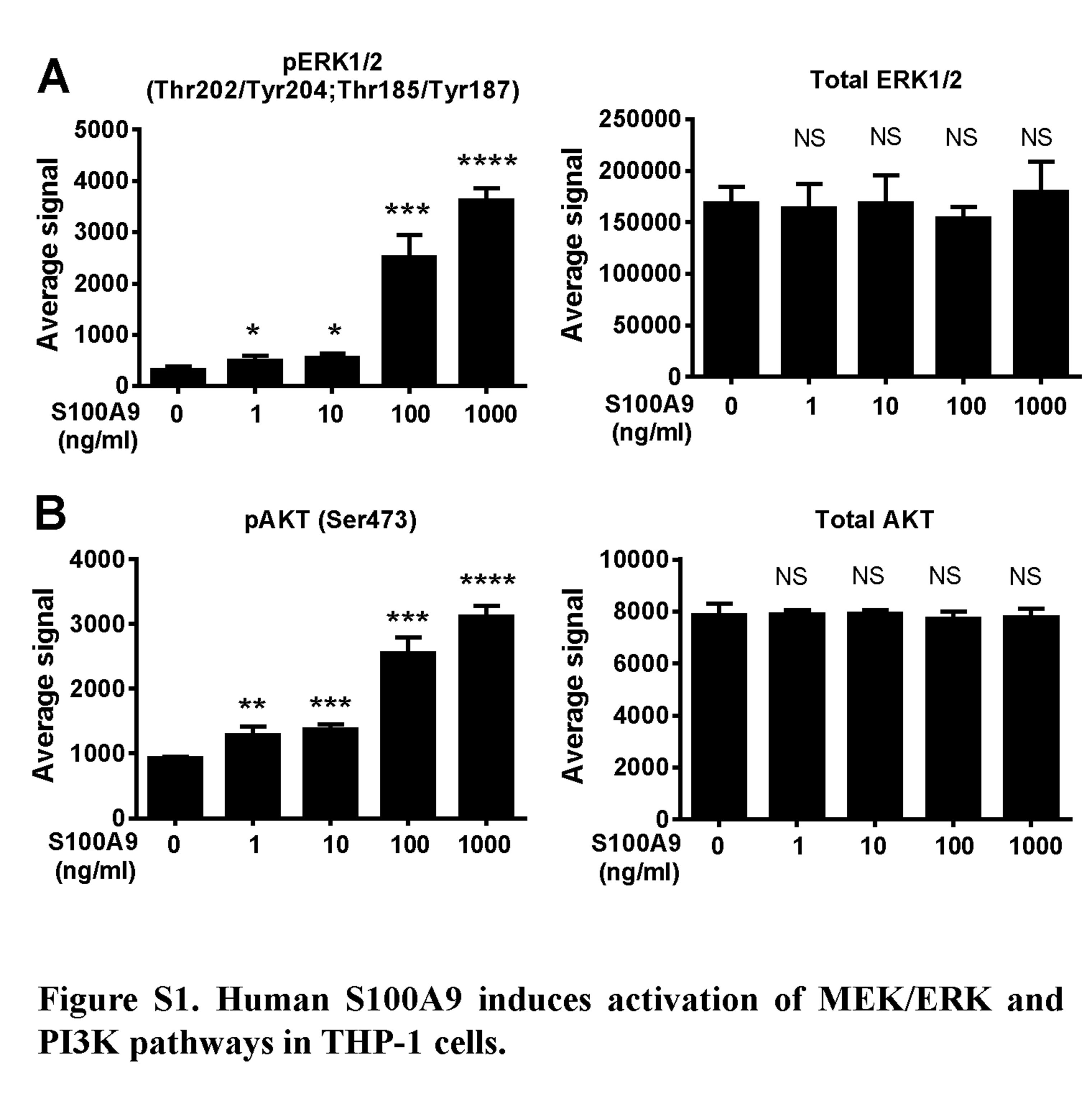

Supplement: S1 Fig — THP-1 cells were treated with S100A9 at indicated concentration for 30 mins, cell lysates were collected, and Phospho ERK1/2 and total ERK1/2 were measured by Phospho(Thr202/Tyr204; Thr185/Tyr187)/total ERK1/2 assay whole cell lysate kit (A), PhosphoAKT and total AKT were measured by Phospho(Ser473) /Total AKT assay whole cell lysate kit (B). Data shown is mean±SD from three independent experiments. *P<0.05, **P<0.01, ***P<0.001 versus control cultures. (TIF) [file pone.0115828.s001.tif]
